# Supplementary material for: Whole Yeast Vaccine Displaying ZIKV B and T Cell Epitopes Induces Cellular Immune Responses in the Murine Model
Source: Pharmaceutics. 2023 Jul 6;15(7):1898. doi: 10.3390/pharmaceutics15071898 (PMC10385271; doi:10.3390/pharmaceutics15071898)
Supplement: Supplementary file 1 [file pharmaceutics-15-01898-s001.zip › pharmaceutics-2414457-supplementary.pdf]

Supplementary Material

Table S1: Secretion of splenic cytokines after *in vitro* re-stimulus. There was no significant stimulus for the production of cytokines IL-2, IL-4, IL-10, IL-17A. Levels measured in pg.mL^{-1}. Values express mean ± standard deviation.

	24h			48h			72h		
**Cytokine**	**nr**	**ENV**	**ENVNS1**	**nr**	**ENV**	**ENVNS1**	**nr**	**ENV**	**ENVNS1**
**IL-2**	440.89 ± 13.51	447.33 ± 5.79	432.00 ± 13.69	421.77 ± 7.05	432.72 ± 8.21	419.19 ± 4.04	410.6 ± 10.02	412.80 ± 2.55	417.42 ± 13.12
**IL-4**	907.44 ± 0.90	905.87 ± 0.09	906.68 ± 0.88	905.67 ± 0.58	905.66 ± 0.70	905.60 ± 0.58	904.47 ± 0.55	904.78 ± 0.51	905.54 ± 0.77
**IL-10**	132.75 ± 8.02	117.48 ± 5.30	130.56 ± 4.11	117.31 ± 6.51	123.21 ± 7.85	121.41 ± 13.85	119.53 ± 12.85	124.0 1± 7.67	124.48 ± 8.65
**IL-17A**	153.59 ± 1.21	150.09 ± 0.96	152.55 ± 3.61	151.35 ± 1.55	156.86 ± 4.12	151.28 ± 7.68	145.75 ± 2.85	146.89 ± 1.91	149.45 ± 5.14

Table S2: Average weight of the mice groups throughout the immunization experiment. Values correspond to mean ± standard deviation.

**GROUPS**	**Weight (g)**		
	**Day 1**	**Day 7**	**Day 21**
***P. pastoris*:nr**	21.66 ± 0.90	22.74 ± 0.99	23.27 ± 1.20
***P.pastoris*:ENV**	22.06 ± 1.63	22.63 ± 1.77	23.71 ± 2.02
***P.pastoris*:ENVNS1**	20.86 ± 0.55	22.03 ± 1.03	22.75 ± 1.01
